# Numerical simulation and field experiment study of the supersonic gas jet subsoiler based on DEM

**DOI:** 10.1371/journal.pone.0328565

**Published:** 2025-08-14

**Authors:** Xia Li, Zhipeng Zhao, Birong You, Xuhui Wang, Tianyu Qi, Hang Zhu, Tao Qin

**Affiliations:** 1 Tianjin Key Laboratory for Advanced Mechatronic System Design and Intelligent Control, School of Mechanical Engineering, Tianjin University of Technology, Tianjin, China; 2 National Demonstration Centre for Experimental Mechanical and Electrical Engineering Education, Tianjin University of Technology, Tianjin, China; Hindustan Institute of Technology and Science, INDIA

## Abstract

To tackle the challenges of high draft resistance, limited subsoiling range, and ineffective subsoiling results in conventional subsoiling methods, this study combines the structure of an air cannon with that of a wing-type subsoiler to design a novel pneumatic blast subsoiling device. First, soil bin experiments were conducted to verify the feasibility of using the air cannon for pneumatic soil fracturing. A soil model was created in EDEM (Engineering Discrete Element Method)based on field conditions to analyze the subsoiler’s force distribution, ensuring the nozzle remains undeformed and the subsoiling process runs smoothly. Subsequent field experiments were conducted to evaluate the subsoiling effect under different working depths, speeds, and air pressures. The experimental results show that, compared with the conventional airfoil-shaped subsoiler, the supersonic gas jet subsoiler achieves optimal performance at a tillage speed of 0.5 m/s and a tillage depth of 380 mm. Under these conditions, the maximum drag reduction rate reaches 16.66%, and the soil disturbance area increases by up to 22.48%, significantly enhancing the drag reduction effect and soil fragmentation efficiency during subsoiling operations. Furthermore, the subsoiling effect was further improved as the working speed decreased and the frequency of air blasts increased, satisfying the subsoiling operation assessment standards. In conclusion, this study offers design insights for the development of innovative agricultural soil cultivation tools by identifying a more efficient new research approach in addition to conventional subsoiling techniques for lowering resistance and energy consumption.

## Introduction

Soil degradation is a global challenge. Due to overuse of machinery, intensive planting, short crop rotations, overgrazing, and poor soil management in agricultural practices, soil compaction has become a significant problem in modern agriculture as China’s degree of mechanization rises [1]. Continuous tillage raises and thickens the plough pan, which hinders gas release in the soil and plant root growth [2]. Once the soil is compacted, natural recovery requires a long process, whereas soil subsoiling can accelerate the soil’s restoration [3]. Subsoiling is an essential element of conservation tillage practices [4]. Performing subsoiling operations on cultivated land can break up the soil plough pan, lower soil bulk density, improve soil structure, increase soil porosity, and enhance nutrient utilization in the soil [5–7]. It can also improve the soil’s water retention and drought resistance, which in turn promotes root growth and boosts crop yield [8,9]. Currently, mechanical subsoilers, such as airfoil-shaped subsoilers and vibrating subsoilers, are the main tools used for soil subsoiling. However, these methods commonly suffer from issues such as poor subsoiling effects, uneven subsoiling depth, and high energy consumption. Although the primary research strategy in this field is on optimizing subsoiler structure to lower energy consumption, it is still unable to satisfy production expectations [10–12].

Pneumatic subsoiler research has steadily increased in the last few years. In order to help with subsoiling, pneumatic subsoilers inject high-pressure gas into the soil to produce jet impacts. This technique can efficiently break the plow pan by lowering tillage resistance and friction between the subsoiler and the soil. Araya et al. investigated how the placement angle of the subsoiler affects the efficiency of the subsoiling process after incorporating a gas injection mechanism at the front of the subsoiler [13]. In order to analyze the characteristics of pneumatic subsoiling and verify its feasibility, Zuo et al. carried out a jetting experiment on soil using a high-pressure gas nozzle [10]. Xi et al. developed a blast-induced soil fracture trajectory equation and designed an orthogonal experiment to analyze the impact of gas jet on soil fracture propagation [14]. The research by Tang et al. and others showed that injecting air into rice paddy soil can reduce N_2_O and NH_3_ emissions, regulate the nitrogen cycle, and enhance the soil oxygen diffusion rate [15]. The aforementioned studies indicate that conducting field experiments on pneumatic subsoiling based on the principle of gas pressure fracturing is feasible. However, researchers have long ignored pneumatic blast subsoiling devices because they lack effective experimental optimization techniques and appropriate novel mechanisms.

Drawing upon pertinent literature and our team’s prior research endeavors, pneumatic subsoilers conventionally position the gas outlet at the tip of the subsoiler blade to exert jet impacts upon the soil [16–19]. During subsoiling operations, the tractor propels the pneumatic subsoiler forward. As tillage progresses, an abundance of soil clods accumulates ahead of the subsoiler, partially obstructing the gas outlet. Consequently, the shockwave impact from the high-pressure gas upon release diminishes, thereby weakening the drag reduction effect of pneumatic subsoiling. Therefore, increasing the airflow velocity and impact force on the soil emerges as a pivotal concern. This enhancement would contribute to lowering fuel consumption and costs, enhancing work efficiency, and carrying substantial scientific significance [20–22]. Xu et al. mounted a Laval nozzle at the outlet of the air cannon, which effectively enhanced the airflow’s impact force without increasing the air mass flow rate [23]. Inspired by this concept, this study endeavors to attain a higher instantaneous airflow impact force by integrating a device capable of generating supersonic airflow with considerable instantaneous impact force, known as the “air cannon.” The air cannon serves as a clearing device that generates high impact force by instantaneously releasing compressed air stored in a pressure vessel. The compressed air contained within the container is discharged into the atmosphere at supersonic speeds (>360 m/s) within a fleeting duration [24]. This device is widely used in conveying pipelines within warehouses, docks, kilns, coal mines, the building materials industry, and chemical engineering, where it plays a pivotal role [23].

**Fig 1:**
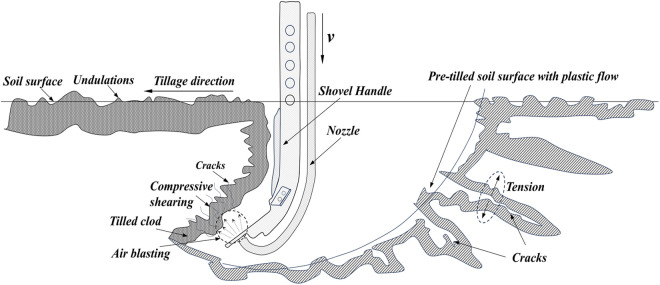
Gas jet subsoiling model.

This study enhances the conventional subsoiler by integrating an air cannon with the airfoil-shaped subsoiler structure and incorporating a Laval nozzle at the gas outlet to enhance the airflow’s impact force. Supersonic gas jet subsoiling experiments are conducted to reduce soil draft resistance and increase the soil disturbance area. A schematic model is shown in Fig 1. The objectives of this study encompass:

I Field soil was collected for soil bin experiments to verify the feasibility of using the air cannon to crack the soil.
II A supersonic gas jet subsoiler was designed and a mathematical model for the air cannon’s pneumatic explosion was established. Numerical simulations using the DEM (Discrete Element Method)method were conducted to ensure that the nozzle of the improved subsoiler would not deform and its performance would not degrade.
III Conducting field experiments to collect relevant data, utilizing draft resistance, soil disturbance area, soil looseness, and soil disturbance coefficient as evaluation indicators to study the working conditions for achieving optimal subsoiling effects.

## Materials and methods

### Soil bin experiment

#### Soil properties.

**Fig 2:**
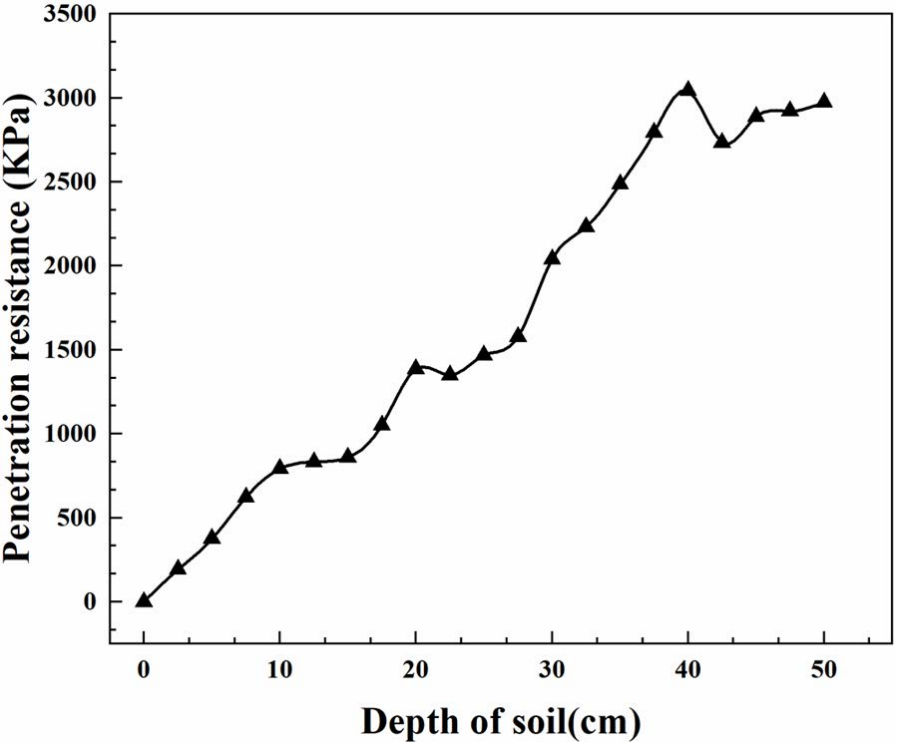
Variation in soil penetration resistance.


The field experiment was conducted at the experimental field located in Xizhaizhuang Town, Jinghai District, Tianjin, China (117.01°E, 38.77°N). The experimental area has a sandy loam soil texture and a flat terrain. Prior to this experiment, corn was the last crop planted, and no subsoiling treatment had been performed on the land. The experimental site was provided by the Agricultural Cooperative of Xizhaizhuang Town, Jinghai District, Tianjin. To investigate subsoiling resistance and test soil parameters, the average soil penetration resistance was measured using a soil compaction instrument at 50 mm intervals, with a depth range of 0–50 cm. The measurement results are shown in Fig 2. Soil moisture content was measured at 12 points, with sampling depths ranging from 10-50 cm. The specific procedure involved utilizing a core sampler (volume: 100 cm²) to collect soil samples. The samples were subsequently transferred to aluminum containers, sealed, and weighed on an analytical balance with a precision of 0.1 mg. Following this, the samples were dried in an oven at 105°C until all moisture had evaporated. Upon drying, the soil was reweighed. The average soil moisture content was determined to be 18.6%. The soil moisture content was calculated using the following equation (1):

$$soil\;moisture\;content\;(\%)=(M{\mathrm{\backslash nolimits}}_{w}-M{\mathrm{\backslash nolimits}}_{d})\times 100\%/\;\;\;M{\mathrm{\backslash nolimits}}_{d}$$ (1)

In the equation, *M*_*w*_ represents the mass of the soil before drying, and *M*_*d*_ denotes the mass of the soil after drying to a constant weight.

#### Soil bin experimental validation of air cannon-induced pneumatic fracturing.

**Fig 3:**
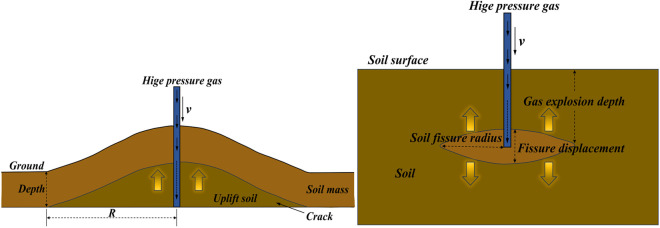
Failure form and mechanism diagram. (A) Soil uplift mechanism; (B) Soil fissure expansion schematic by shear damage.

Currently, there has been no research conducted domestically or internationally on using the high-impact airflow generated by the air cannon for pneumatic subsoiling on cultivated land, and the effectiveness of subsoiling remains unknown. To test the soil fracture effects of the airflow produced by the air cannon, a feasibility study was conducted using a right-angle pulsed air cannon in a soil bin experiment before designing the supersonic gas jet subsoiler. The failure modes and mechanisms of the soil are shown in Fig 3A and 3B. The soil utilized in these experiments was sourced from the experimental field located in Xizhaizhuang Town, Jinghai District, Tianjin, China. A soil bin, measuring 0.8 m × 0.8 m × 0.5 m and constructed from stainless steel plates. To ensure the soil parameters in the soil bin closely match the field soil conditions, a layered approach was used to collect soil from different depths of the field based on its actual structure. The soil was then backfilled, compacted, and leveled with a wooden board. Soil moisture content and compaction were subsequently measured until the values in the soil bin were comparable to those of the field soil [10,14]. Due to safety concerns and experimental constraints, a small right-angle pulse air cannon (rated working pressure of 0.8 MPa, volume of 20 L) was used in the experiments. The air cannon was mounted on a test frame (1.2 m × 0.7 m × 0.7 m) to apply air pressure splitting to the soil. The testing pressures were 0.4 MPa, 0.6 MPa, and 0.8 MPa, with a testing depth of 30 cm.

**Fig 4:**
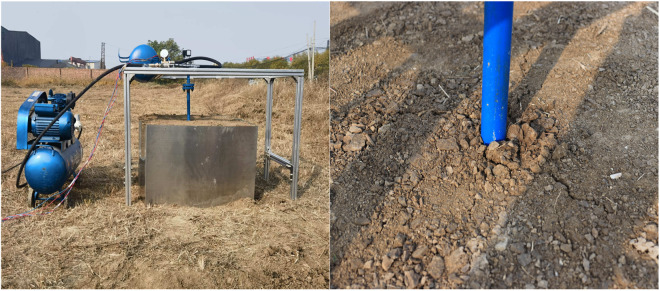
(A) Soil bin experiment site; (B) Air cannon cracking effect^‌‌^.

As per the study conducted by King et al. [25], the primary factors influencing soil fracturing are the overburden stress and the tensile strength of the soil layers. When the jetting point is situated shallowly and the overburden pressure is relatively low, the soil undergoes notable uplift. Consequently, this study evaluated the viability of pneumatic blast by examining both the magnitude of surface uplift and the distance of crack propagation. To validate the effectiveness of the gas jet, the surface uplift of the soil was meticulously measured using a depth camera (DELL D435i) [26]. Fig 4 illustrates the soil fragmentation effect after the pneumatic blast in the soil bin experiment.

### EDEM simulation

#### Establishment of the discrete element model for soil.

Over the past few decades, soil analysis methods have garnered significant interest from researchers. The application of numerical simulation techniques to model soil-tool interactions has decreased the expenses associated with field testing [27,28]. In this study, the Discrete Element Method (DEM) was employed to model soil particles. The method represents the soil as a composition of discrete soil particles and soil inter-particle voids, allowing the soil particles to deform, rotate, and move. Additionally, the soil voids can be compressed, slid, or separated, while the soil particles themselves can also deform [29]. The interaction between particles is calculated using the contact model and is governed by physical laws. After computing all the forces acting on the particles, their position and direction are determined through the integration of Newton’s second law of motion [30,31]. In this study, the soil is also considered a discontinuous, discrete medium. The soil is modeled in layers based on actual conditions, allowing for the simulation of soil fracturing during subsoiling and the nonlinear stress characteristics of the subsoiler in the field.

**Fig 5:**
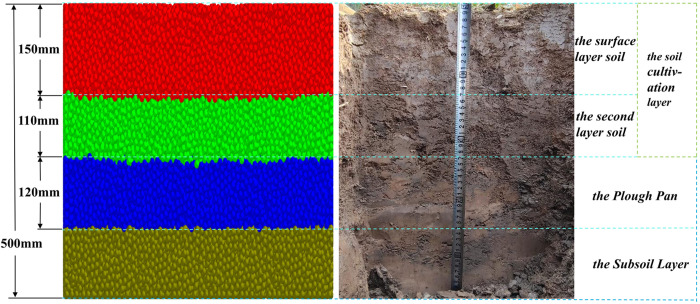
(A) The soil model in EDEM; (B)The actual condition of the field soil.

**Fig 6:**
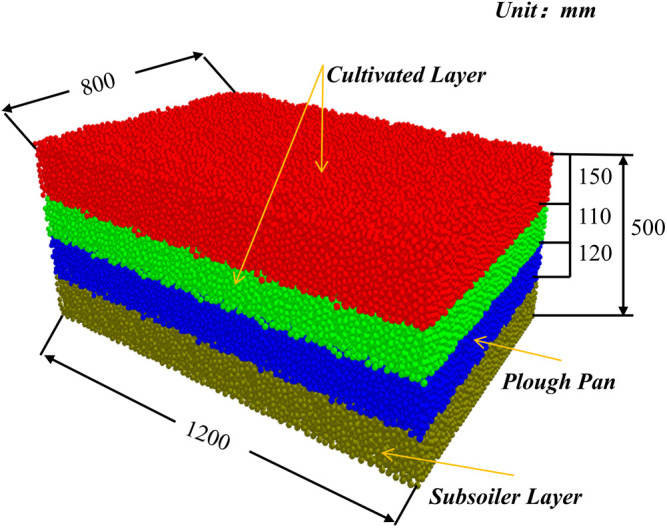
The soil bin and soil stratification established by EDEM.

For soil that has undergone long-term cultivation, various tillage methods impact different soil layers at varying depths, leading to notable variations among the tillage layers [32]. To gain a deeper understanding of the alterations in soil condition and soil structure, this study first establishes an EDEM simulation model based on the actual field soil conditions (Fig 5). The initial layer, which is 150 mm deep, represents the surface soil, while the subsequent layer, measuring 110 mm in depth, constitutes the second tillage layer (colored in red and green). The third layer, with a thickness of 120 mm, represents the plough pan (colored in blue), and the fourth layer, also 120 mm thick, represents the subsoil layer (colored in brown). Based on the recommendation of Wang et al, the particle radius is set at 7 mm to ensure computational accuracy with reduced computational load [31]. The particle contact radius is set to 1.1 times the particle radius [33]. The designed soil model is shown in Fig 6, with dimensions of 1200 mm (length) × 800 mm (width) × 500 mm (depth).

In this study, the parameters necessary for constructing the soil model were obtained through a review of relevant literature and actual measurements [34–37]. The EDEM analysis was conducted using the Hertz-Mindlin with JKR model, which is a cohesive contact model that provides high-precision simulations of the adhesive forces between particles [38]. The adhesive force in the Hertz-Mindlin with JKR model depends on the surface energy of the generated soil particles. By inputting the corresponding soil surface energy into the model, the adhesion between soil particles can be effectively minimized [12]. The surface energy between soil particles was selected based on the findings reported by Hu et al. [39], as the experimental soil samples share the same soil type and moisture content. In this study, the JKR surface energy was set at 6 J/m². The values of the EDEM contact and material parameters are shown in Table 1.

**Table 1:** Basic parameters of the EDEM used in the simulations.

Parameters	Soil layers			
	The surface layer soil	The second layer soil	The Plough Pan	The Subsoil Layer
	0-150 mm	150-260 mm	260-380 mm	380-500 mm
Density of soil particles (kg/m^3^)	1800	1850	1880	1850
Poisson’s ratio of sand	0.3	0.3	0.3	0.3
Shear modulus of sand (Pa)	1.02e + 8	1.03e + 8	1e + 8	1.02e + 8
Particle radius (mm)	7	7	7	7
Coefficient of restitution, soil-soil	0.53	0.49	0.46	0.45
Coefficient of static friction, soil-soil	0.5	0.45	0.45	0.38
Coefficient of rolling friction, soil-soil	0.34	0.3	0.27	0.25
Density of 65Mn steel (kg/m^3^)	7865	7865	7865	7865
Poisson’s ratio of steel	0.3	0.3	0.3	0.3
Shear modulus of steel (10^10^)	7.9	7.9	7.9	7.9
Coefficient of restitution, soil-steel	0.28	0.25	0.25	0.25
Coefficient of static friction, soil-steel	0.50	0.48	0.45	0.4
Coefficient of rolling friction, soil-steel	0.32	0.28	0.27	0.24

#### EDEM analysis of the effect of nozzle on subsoiler performance.

Due to the rigid structure of the nozzle installed at the rear of the supersonic gas jet subsoiler, where the nozzle diameter is narrower than the width of the subsoiler shank and positioned subsequent to it, the friction between the nozzle and the soil, as well as the particle accumulation between the nozzle and the shank, remains unpredictable. Given the challenges associated with directly measuring the stress distribution on the subsoiler components during actual tillage operations, and to eliminate interference factors and prevent nozzle deformation that might compromise subsoiler performance, utilizing EDEM software for stress analysis during subsoiler operation has become the optimal alternative approach.

The plough pan in farmland soil typically varies at depths around 300 mm below the surface. However, excessive subsoiling depth can lead to water resource wastage during irrigation. According to the Chinese standard (JB/T 10295−2014), the subsoiling depth should not fall below 250 mm. Taking into account the actual soil stratification in the field, subsoiling operations were performed at depths of 250 mm above the plough pan, 300 mm near the plough pan, and 380 mm beneath the plough pan. Furthermore, literature reports indicate that the typical working speed of subsoiling operations ranges from 0.5 m/s to 2.5 m/s [40]. To ensure that the improved subsoiler can adapt to a diverse array of operating conditions, this study selected subsoiling speeds of 0.5 m/s, 0.83 m/s, and 1.8 m/s for EDEM simulation [31,35,41].

Due to variations in simulation parameters and computer configurations, accurately estimating the time required for EDEM simulations can be challenging. A key parameter in the EDEM simulation is the Rayleigh time step, which can be calculated using Equation (2) [42]. In this study, a typical simulation time step of 0.2TR (20%) has been deemed appropriate and meets the simulation requirements for most cases [35]. The Rayleigh time step was set to 0.00010119 s, with a data saving interval of 0.01 s. Once the parameters have been set up, both the wing-type subsoiler and the supersonic gas jet subsoiler were imported into the EDEM software. Subsequently, the forces acting on these two subsoilers and the variation of their draft resistance were analyzed under conditions where no airflow affected the plough pan.


$$T{\mathrm{\backslash nolimits}}_{R}=\frac{\pi R(\frac{\rho }{G})}{\left(0.1631v+0.8766\right)}$$ (2)

In the equation, R represents the particle radius, ρ denotes the density, G is the shear modulus, and *v* is the Poisson’s ratio.

### Field experiments

#### Design and analysis of structural parameters for the supersonic gas jet subsoiler.

**Fig 7:**
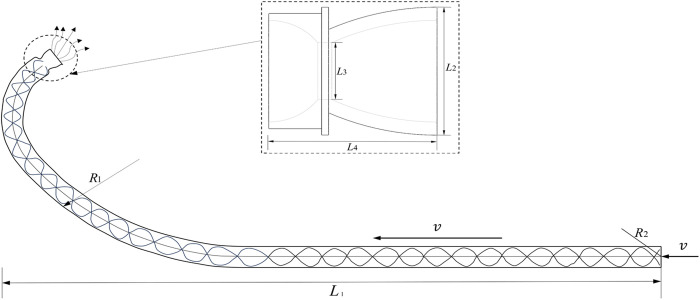
Schematic diagram of the airflow passage.

This study enhances the wing-type subsoiler design by opting for a shank shaped as a circular arc in accordance with the Chinese standard (JB/T 9788−1999). Each component of the subsoiler is crafted from high-strength 65Mn steel, with the parameters drawn from e previously published data [16,43,44]. In past field tillage experiments involving pneumatic subsoilers, PVC pipes were employed to facilitate high-pressure airflow injection into the subsoiler tip. However, given PVC’s inability to withstand the high-impact airflow generated by the air cannon, this study instead utilizes robust materials for the nozzle construction. The designed nozzle has a curvature radius R_1_ of 320 mm, an outer diameter R_2_ of 23 mm, a wall thickness of 1.5 mm, and an overall length L_1_ of 700 mm. It connects to the air cannon nozzle via a flange and is mounted behind the shank. Notably, the nozzle diameter is narrower than the shank’s width, ensuring that the shank bears all resistance, thereby preventing excessive stress on the nozzle during operation and safeguarding against deformation. This configuration ensures a seamless subsoiling process. A schematic illustrating the airflow explosion pathway is presented in Fig 7.

Zou et al.‘s study reveals that the maximum stress is concentrated at the bottom of the subsoiler [45]. Therefore, in this study, the nozzle is welded to the bottom of the subsoiler tip. The outer diameter of the nozzle L_2_ is 20 mm, accompanied by a wall thickness of 1 mm and a throat diameter L_3_ of 9 mm. The overall length L_4_ of the nozzle is 25 mm. To further accelerate the airflow velocity prior to its collision with the plough pan, enhancing its impact force, a Laval nozzle is incorporated.

Under the conditions of one-dimensional steady-state isentropic uniform flow, and assuming negligible pressure fluctuations within the high-pressure air tank during the exhaust phase, the exhaust impact force at the air cannon’s outlet aligns with the theoretical maximum impact force. The air density in the air cannon can be calculated using the ideal gas state equation (3), as outlined in the subsequent formula:


$$P=\rho {\mathrm{\backslash nolimits}}_{o}RT$$ (3)

$$F=A[(P{\mathrm{\backslash nolimits}}_{o}-P{\mathrm{\backslash nolimits}}_{e})+\rho {\mathrm{\backslash nolimits}}_{o}V{\mathrm{\backslash nolimits}}_{e}^{2}]$$ (4)

In equation (4),$\rho_{O}$ represents the air density(kg/m^3^);R is the gas constant (J/(kmol·K));T is the absolute temperature(K);A is the cross-sectional area of the Laval nozzle outlet(mm^2^);*P*_***O***_ is the rated working pressure of the air cannon; *P*_***e***_ is the environmental pressure at the nozzle outlet(MPa); and *V*_***e***_ is the gas ejection velocity(m/s).

Based on the research conducted by Jia et al., the length of the injection pipeline, coupled with the incorporation of elbows, can lead to a reduction in the impact force of the gas flow [46]. The attenuation rate of the injection pipeline curvature is taken as 10%. Through calculations, the air density $\rho_{O}$ is determined to be 9.35 kg/m^3^, and the gas explosion impact force F exerted by the air cannon on the soil amounts to 657.45 N.

Beyond the requirement of a significant pressure difference between the inlet and outlet of the nozzle to generate supersonic airflow, the pipeline must also possess a geometry that is conducive to continuous pressure reduction, expansion, and acceleration of the airflow. Specifically, the pipeline should first undergo a gradual convergence to accelerate the subsonic flow, reaching the speed of sound at the throat, followed by a gradual expansion to further propel the airflow into a supersonic state. Notably, the airflow in the Laval nozzle is capable of achieving supersonic airflow speeds [47]. The formula for calculating the Mach number is: [23]


$$M=\sqrt{\left(\left(\frac{P{\mathrm{\backslash nolimits}}_{o}}{P{\mathrm{\backslash nolimits}}_{e}}\right){\mathrm{\backslash nolimits}}^{\frac{k-1}{k}}-1\right)\times \frac{2}{k-1}}$$ (5)

Since the flow of the working medium is assumed to be isentropic, the gas injection velocity at the air cannon outlet can be calculated using the isentropic flow equation. The formula for the velocity of the gas at the nozzle exit is:


$$V{\mathrm{\backslash nolimits}}_{e}=\sqrt{kRT\left[1+\frac{K-1}{2}M{\mathrm{\backslash nolimits}}^{2}\right]{\mathrm{\backslash nolimits}}^{-1}}$$ (6)

In equation (6), k represents the specific heat ratio, and M represents the Mach number, both of which are dimensionless quantities.

The calculated Mach number M = 2.01, and at a standard temperature of 27°C, the gas injection velocity *V*_***e***_ = 416.92m/s.

In reference to the working process of the air cannon, its exhaust process is reconfigured into a nozzle with a stable and infinite air supply. The air-blasting process relies on the instantaneous expansion of high-pressure air, occurring over a fleeting duration with minimal heat exchange, thereby qualifying it as an adiabatic expansion process. According to the principles of engineering thermodynamics [47], the work accomplished by the adiabatic expansion of the air cannon tank, subsequent to the accumulation of pressure energy, can be computed using the formula provided below:


$$W=\frac{1}{k-1}P{\mathrm{\backslash nolimits}}_{o}V\left[1-\left(\frac{P{\mathrm{\backslash nolimits}}_{e}}{P{\mathrm{\backslash nolimits}}_{o}}\right){\mathrm{\backslash nolimits}}^{\frac{k-1}{k}}\right]$$ (7)

In equation (7), W is the work accomplished by the adiabatic expansion of the high-pressure air tank(J); V is the volume of the air cannon(m³).

**Fig 8:**
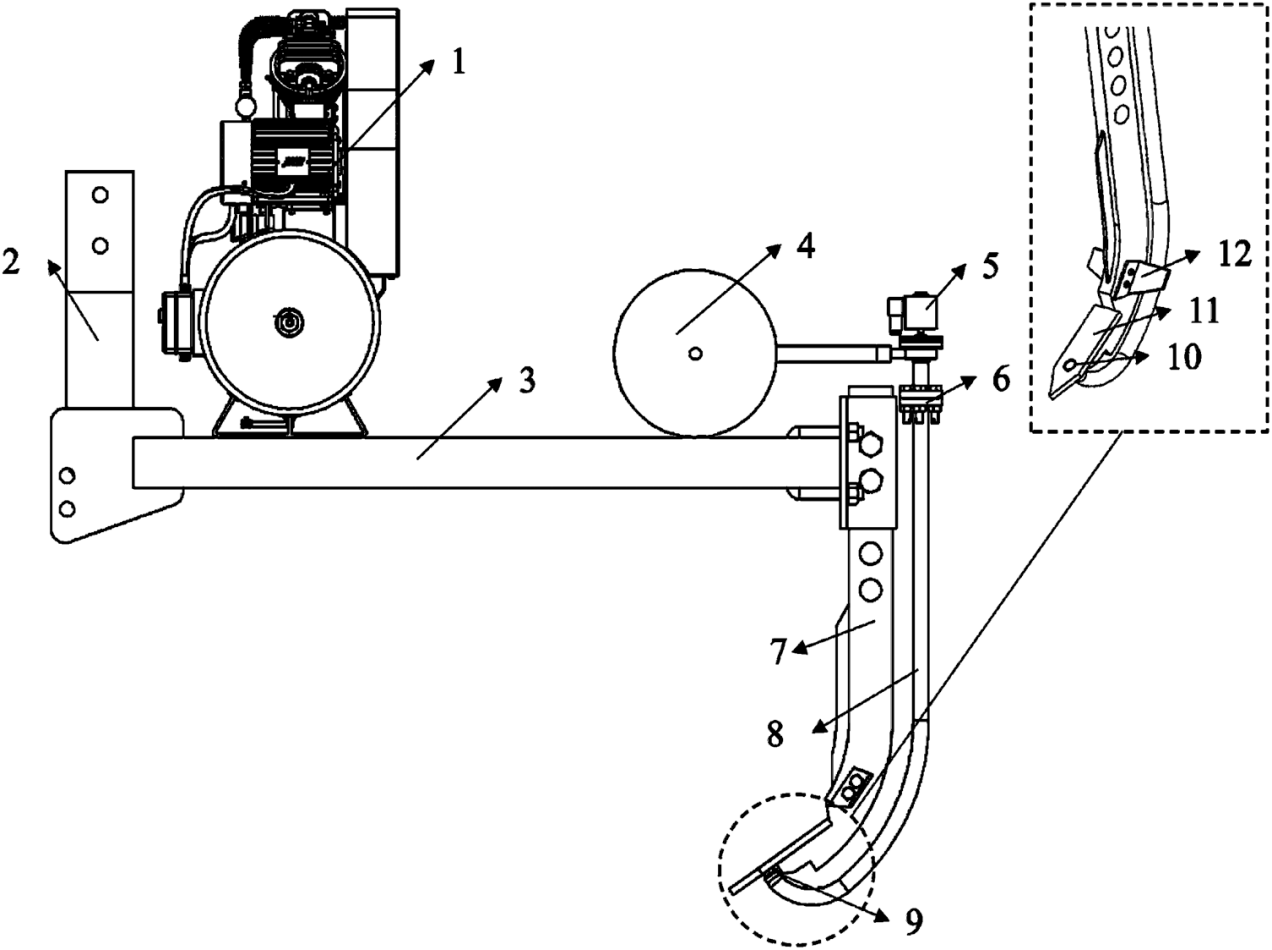
The schematic diagram of the supersonic gas jet subsoiler structure. (1) Air compressor; (2) Traction suspension; (3) Frame; (4) Air cannon; (5) Pulse solenoid valve; (6) Flange plate; (7) Subsoiler; (8) Drain gas pip; (9) Laval nozzle; (10) Air outlet; (11) Shovel tip; (12) Wing.

The volume of the air cannon is known to be 20L. Substituting into equation (7), the work accomplished by the adiabatic expansion of the air cannon is calculated as W = 1.79 × 104 J.A schematic diagram of the subsoiler structure is shown in Fig 8.

#### Testing equipment.

**Fig 9:**
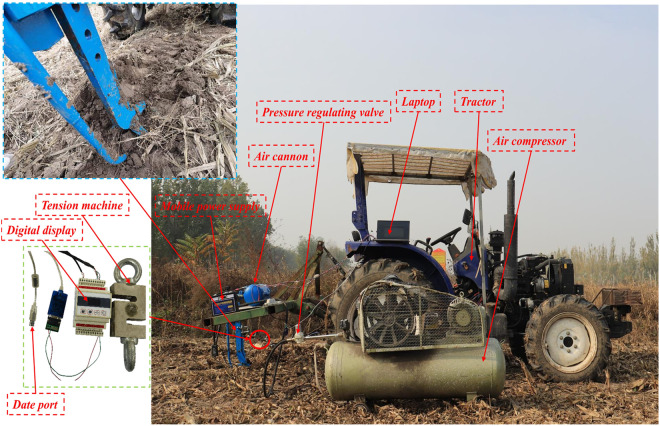
Supersonic gas jet subsoiler experiment.

Field experiments serve as a crucial means to validate the actual effects of soil tillage and assess the influence of improved soil tillage equipment on soil structure and draft resistance. The experimental setup encompasses a range of equipment, including the supersonic gas jet subsoiler, a right-angle pulsed air cannon, an air compressor, a generator, a subsoiler frame, a tractor, an S-type digital tension sensor (manufactured by Dayang Sensor System Engineering Co, Ltd., located in Bengbu, Anhui Province, China; with a measurement range of 0-5T), an insert plate, a tape measure, six shackles (M10), and a laptop computer. The subsoiler and tension sensor are securely connected through shackles. As the tractor advances, it propels the subsoiler, while the tension sensor, linked to the laptop, facilitates the uploading of tension data. A schematic representation of the pneumatic subsoiling experiment site is depicted in Fig 9. The technical parameters of the main experimental equipment are shown in Table 2.

**Table 2:** Main structure and performance parameters of the machine.

Items	Value
Overall dimensions of the air cannon(mm)	400 × 220 × 220
Air jet characteristics	Pulsed
Rated power of the air compressor(kW)	4
Rated pressure of the air compressor(MPa)	0.8
Air compressor air displacement (m^3^/min)	0.67
Overall dimensions of the generator(mm)	720 × 570 × 590
Rated power of the generator(kW)	12
Rated voltage of the generator (V)	220/380

#### Field experiment procedure.

Numerous factors have a considerable influence on the draft resistance and soil disturbance conditions of the subsoiler, including its shape, working depth, forward speed, soil condition, and air pressure [48]. In this study, a comprehensive factorial experiment was conducted to evaluate the tillage performance of the supersonic gas jet subsoiler using various combinations of operating speed, working depth, and air pressure. At the same time, the unoptimized wing-type subsoiler was used as the experimental control group (No pressure). The experimental design is shown in Table 3, comprising a total of 36 test groups. Each group was repeated three times under identical field conditions, with consistent operating parameters maintained throughout the repetitions.

**Table 3:** Test plan table.

Test number	Deep tillage (cm)	Speed (m/s)	Test number	Deep tillage (cm)	Speed (m/s)	Test number	Deep tillage (cm)	Speed (m/s)	Pressure type
F_1_	25	0.5	F_4_	30	0.5	F_7_	38	0.5	No pressure
									Pressure I I
									Pressure II
									Pressure III
F_2_		0.83	F_5_		0.83	F_8_		0.83	No pressure
									Pressure I
									Pressure II
									Pressure III
F_3_		1.8	F_6_		1.8	F_9_		1.8	No pressure
									Pressure I
									Pressure II
									Pressure III

^a^No pressure value is 0MPa(The wing-shaped subsoiler), Pressure I value is 0.4MPa, Pressure II value is 0.6MPa, and Pressure III value is 0.8MPa.

Based on the surface conditions of the experimental field, the subsoiler height was adjusted using the three-point suspension device at the rear of the tractor. Once the tractor applied draft force, the subsoiler was positioned at the predetermined working depth. The air compressor was then activated to inflate the air cannon, and the pressure regulator was used to adjust the air pressure to the required working pressure. The high-pressure gas ejected from the air cannon was directed through the Laval nozzle and sprayed onto the plough pan around the tip of the subsoiler. Under the action of the high-impact airflow, the plough pan expanded, underwent shearing, became loosened, and formed cracks. The high-pressure gas then penetrated the cracks, lifting the soil. The tractor then towed the supersonic gas jet subsoiler forward, thereby completing the subsoiling operation.

#### Evaluation indicators of subsoiling effect.

**Fig 10:**
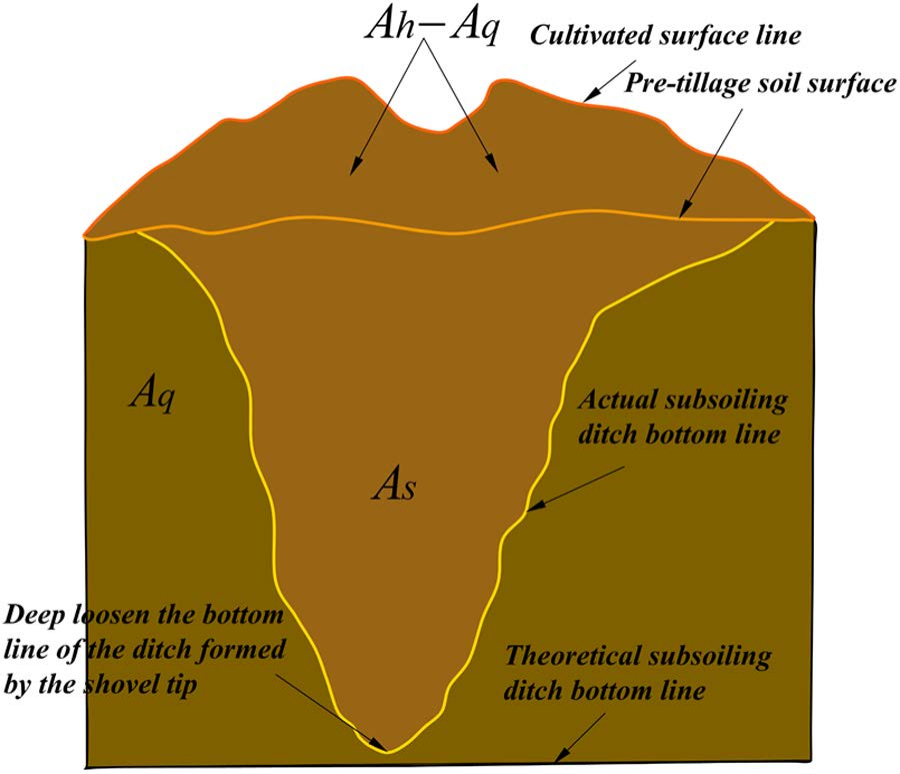
Soil disturbance cross-sectional model.


Subsoiling efficiency is a commonly employed metric to gauge the overall impact of a subsoiler. Researchers typically utilize the soil disturbance area and draft resistance as indicators to evaluate subsoiling efficiency [36,49]. This study utilizes the destructive effect of the subsoiler on the plough pan and employs evaluation metrics such as draft resistance, disturbed area, soil looseness, and soil disturbance coefficient to characterize the subsoiling effects. During the experiments, each test was repeated three times. The draft resistance of the subsoiler was determined by calculating the average subsoiling resistance over a stable travel distance of 30 m, which was used as the final experimental result. To evaluate the soil disturbance effect of the supersonic gas jet subsoiler, the disturbed soil within the furrow was removed in the stable travel zone after subsoiling, and the disturbed area was measured using the insert plate method [40]. After obtaining the disturbed area, a further quantitative evaluation of the soil disturbance characteristics was conducted. The soil looseness and soil disturbance coefficient were determined by analyzing the cross-sectional area from the soil surface before tillage to the bottom of the furrow after subsoiling (Fig 10). The disturbed ridge cross-section was defined by the accumulated soil contour at the surface, and a fitted curve of the subsoiling disturbance profile was obtained using the coordinates of the ridge and pit shapes. The formulas for calculating soil looseness and soil disturbance coefficient are as follows:

$$P=\frac{A{\mathrm{\backslash nolimits}}_{h}-A{\mathrm{\backslash nolimits}}_{q}}{A{\mathrm{\backslash nolimits}}_{q}}\times 100\;\%$$ (8)

In the formula ——P represents the soil looseness

A_q_——The cross-sectional area from the soil surface to the theoretical bottom of the furrow before tillage.

A_h_——The cross-sectional area from the soil surface to the theoretical bottom of the furrow after tillage.


$$y=\frac{A{\mathrm{\backslash nolimits}}_{s}}{A{\mathrm{\backslash nolimits}}_{q}}\times 100\;\%$$ (9)

In the formula

y——The soil disturbance coefficient

A_s_——Cross-sectional area of soil disturbance

## Results and discussion

### Soil uplift and crack propagation distance

**Fig 11:**
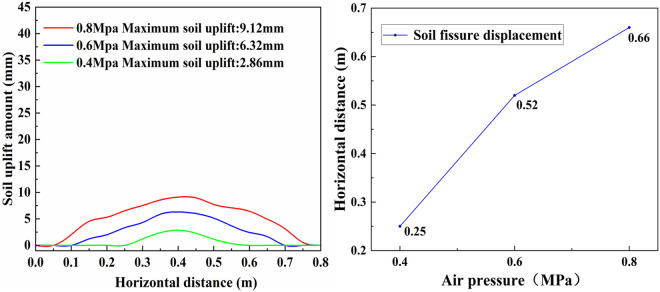
(A) soil surface uplift; (B) Soil fissure displacement.

Preliminary experiments revealed that, when the air cannon pressure within the tank fell below 0.4 MPa, no discernible alterations were noted on the soil surface. As depicted in Fig 11A, the maximum soil surface uplift was recorded at the center of the air blast. The uplift exhibited a gradual decline as the horizontal distance increased, ultimately diminishing to zero with further horizontal displacement. Furthermore, since the airflow was directed vertically downward, the experiment revealed that at an air pressure of 0.8 MPa, the soil around the nozzle was ejected upward, while the soil located beneath the blast center subsided by 5 cm.

The air blast emitted from the air cannon increased the air content within the soil, leading to an expansion in soil volume and consequently, soil surface uplift. However, as the horizontal distance increased, the air pressure decayed to the threshold level necessary for initiating cracks in the soil, thereby terminating crack propagation and halting further surface uplift. A comparative analysis reveals that as the air pressure increased, both the soil surface uplift and crack propagation distance increased accordingly. At the rated working pressure of the air cannon (0.8 MPa), the maximum soil surface uplift and the farthest crack propagation distance were achieved(Fig 11B).Therefore, when the pneumatic blast pressure exceeds the soil’s air-splitting force, a higher blast pressure promotes the expansion of fractures, aligning with the experimental findings. Similar to the yield limit in plastic mechanics, where the external force on a material exceeds its elastic limit, stress ceases to increase but continues to induce plastic deformation [14]. Although the soil surface cracks, upon reaching their expansion limit with increasing horizontal distance, did not significantly propagate further, the cracks generated inside the soil increased its porosity. This aligns with the goal of subsoiling the soil without disturbing the tillage layer. The analysis of the soil bin experiment underscores the effectiveness of utilizing the air cannon for air pressure cracking and its feasibility for subsoiling applications. Therefore, the design of a supersonic gas jet subsoiler was carried out, and its actual subsoiling effect was analyzed through field experiments.

### Performance of the subsoiler in EDEM simulation

**Fig 12:**
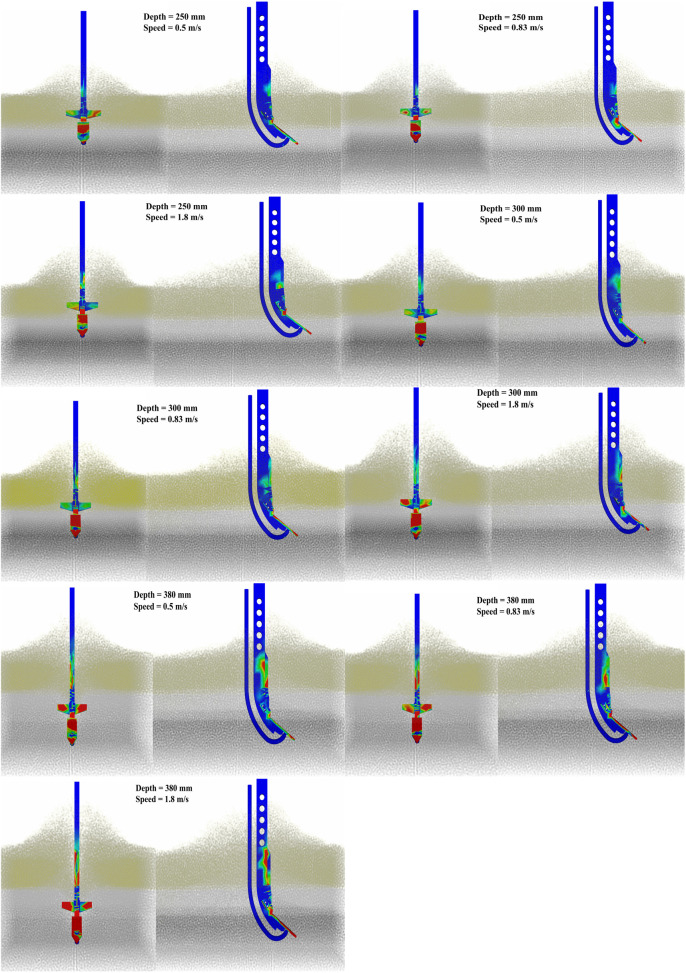
The force distribution diagram of the subsoiler under different working depths and speeds.

The force distribution diagram depicted during subsoiling operations (Fig 12) provides a situation into the stress conditions experienced by various components of the subsoiler. Through simulations of operational forces under diverse working conditions, it becomes evident that the tip of the subsoiler bears the heaviest and most concentrated force, rendering it susceptible to damage. As the depth and speed of operation increase, the forces acting upon the shank and wings increase notably, achieving a more uniform force distribution.

The simulation results demonstrate that the nozzle was not subjected to significant loading during tillage, and its mechanical response is negligible.This is because, during tillage, the tip, winged share, and the curved section of the shank shear and compress the soil under the draft force, causing it to be lifted, fragmented, and displaced. Consequently, a soil ridge forms on the surface, which is subsequently broken down by the shearing action of the shank’s straight section, causing soil particles to accumulate on both sides. Furthermore, the presence of wings exerts pressure and disturbance to both the plough pan soil and the soil adjacent to the tip. This leads to soil loosening and expansion at an angle to the sides, augmenting lateral soil disturbance [9]. This mechanism not only guides soil particle movement but also transforms chaotic soil disturbance into a more orderly motion, reducing the contact area and adhesion between the soil particles and the nozzle, ultimately decreasing the friction.

**Fig 13:**
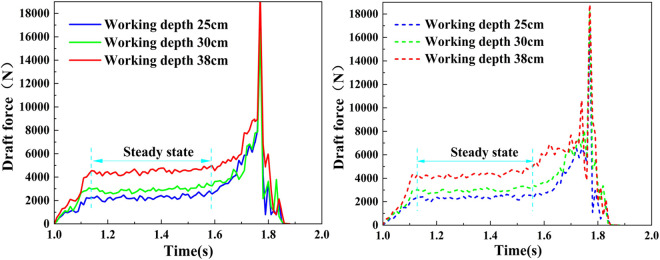
The draft force of the subsoiler at varying working depths when the working speed is 1.8 m/s. (A) Supersonic gas jet subsoiler; (B) Wing-type subsoiler.

EDEM can monitor the horizontal forces acting upon the geometric configuration. This feature is specifically employed to oversee the draft resistance along the virtual subsoiler’s length throughout the simulation process. Taking the working speed of 1.8 m/s as an example, a comparison was conducted between the draft resistance exerted by the supersonic gas jet subsoiler and the wing-shaped subsoiler at varying depths. It was observed that the draft resistance trends of the two machines became consistent. As shown in Fig 13, when the subsoiler enters the soil bin, the draft resistance experiences a swift surge and then fluctuates to a stable value during the steady operation phase. However, due to the spatial constraints of the soil bin structure, the draft force’s stabilization period is relatively brief. As the subsoiler approaches the boundary of the soil bin, a large accumulation of particles occurs at the boundary. This boundary effect triggers a rapid surge in the draft resistance, culminating in a prominent “spike” at the end of the stroke. This aligns with the findings reported in the study conducted by Li et al. [50].

**Fig 14:**
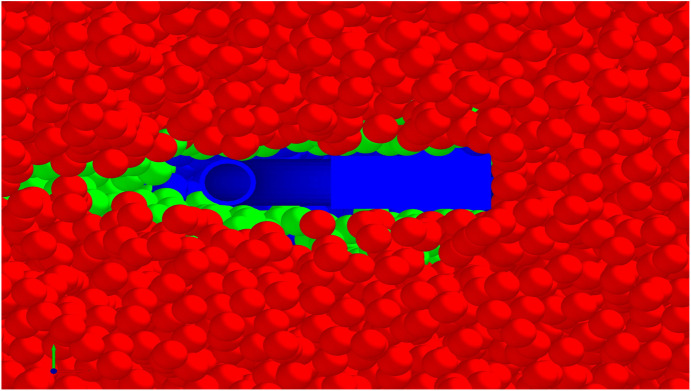
The top view of the subsoiler in the simulation process.

The analysis (Table 4) focused on the average value of the draft force curve during the stable phase (between 1.1s and 1.58s), which corresponds to the midpoint of the soil bin. For tillage depths varying between 250 mm and 380 mm, the average draft resistance error between the supersonic gas jet subsoiler and the wing-shaped subsoiler ranged from 80.96 N to 109.08 N, with a maximum relative error of 4.82%. The error is relatively insignificant and falls within an acceptable threshold. In summary, the analysis shows that, following the installation of the nozzle on the supersonic gas jet subsoiler, the friction force between the nozzle and the soil remains minimal, and there is no accumulation of soil particles between the nozzle and the shank (Fig 14). This prevents deformation of the nozzle during the tillage process. Therefore, the installation of the nozzle does not lead to a significant increase in the draft resistance or a decline in the subsoiler’s performance.

**Table 4:** Average draft force of the two subsoilers at different tillage depths. (unit:N).

Tillage depth (mm)	Supersonic gas jet subsoiler	Airfoil subsoiler	Relative error
250	2261.08	2370.16	4.82%
300	2920.34	3004.99	2.9%
380	4435.62	4354.66	1.86%

### Analysis of field trial results

#### Draft resistance analysis in field trials.

**Fig 15:**
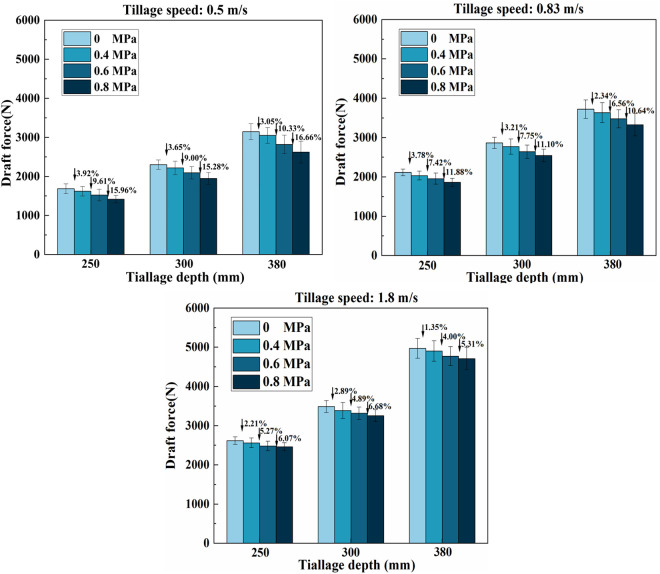
Average draft resistance at different speeds in field experiments. (A) 0.5m/s; (B)0.83m/s; (C)1.8m/s.

Fig 15 illustrates the average draft force during the stable operational phase of the subsoiler in the field experiment. As depicted in the figure, regardless of the operating air pressure, the draft resistance of the supersonic gas jet subsoiler is significantly lower than that of non-explosive subsoiling, clearly demonstrating the drag-reducing effect of explosive subsoiling. However, the drag reduction effect fluctuates based on various factors, including working speeds, depths, and air pressures. When the working speed is set at 0.5 m/s, the working depth is 380 mm, and the working pressure is 0.8 MPa, the maximum drag reduction rate is achieved. Compared with the winged subsoiler, the draft force of the supersonic gas jet subsoiler is reduced by 16.66%.

The experimental results show that the draft force escalates in tandem with an increase in both working depth and speed. At a constant working depth, the draft force augments as the working speed rises. Conversely, under identical working conditions of depth and speed, the drag reduction rate intensifies with an increase in air pressure, leading to a corresponding decline in the draft force. According to Equation (2), as the air pressure increases, the gas velocity accelerates, resulting in a more strong impact force. With the increased pressure within the air cannon tank, the gas jet generated during the instantaneous explosion exerts a higher impact force on the plough pan, causing it and adjacent soil layers to crack and loosen. Subsequently, the subsoiler traverses the working area with a diminished draft force. During the subsoiling process, no deformation of the nozzle under force is observed, which is consistent with the EDEM simulation results.

During the experiment, it was noted that when the supersonic gas jet subsoiler moved at a speed of 1.8 m/s, regardless of the operating air pressure and tillage depth, the reduction rate of tillage resistance was lower compared to the rates observed at speeds of 0.5 m/s and 0.83 m/s. The resistance reduction rate decreased as the working speed increased, potentially attributed to the charging time of the air cannon. Throughout the tillage process, the air compressor consistently supplies high-pressure air to the air cannon. Once the air cannon impacts the soil with the gas jet, the internal pressure of the air tank drops instantaneously, requiring a specific timeframe to recharge the tank pressure back to the operating level for the subsequent blast. Since the charging speed of the air cannon is determined by the air displacement of the air compressor—the greater the air displacement, the faster the charging speed—an increase in working speed reduces the frequency of soil impacts within the same tillage distance, thereby decreasing the draft resistance reduction rate. Therefore, a lower working speed of the supersonic gas jet subsoiler results in more frequent impacts of the air cannon on the soil. This translates to a higher degree of soil loosening around the plowshare and air blast area, generating more cracks and consequently reducing the draft force of the subsoiler.

#### Analysis of soil disturbance effects.

**Fig 16:**
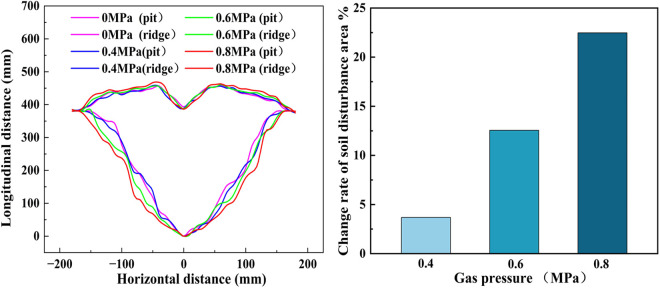
Gas explosion subsoiling drag reduction effect. (A) Variation of soil disturbance area; (B)Variation rate of soil disturbance area.

To date, the literature has failed to document any research exploring the correlation between working speed and the soil disturbance area in the context of soil subsoiling studies. Consequently, this study employs the soil disturbance area, soil looseness, and soil disturbance coefficient at the optimal drag reduction working speed of 0.5 m/s to evaluate the subsoiling effect. Using a plowshare working depth of 380 millimeters as a case in point, Fig 16A illustrates the disturbance patterns of pits and ridges after subsoiling. It can be clearly seen that the supersonic gas jet subsoiler significantly enhances the disturbed area of the soil. Moreover, as the working air pressure increases, so does the soil disturbance area. When the working air pressure is 0.8 MPa, the soil disturbance area experiences the most pronounced increase, as depicted in Fig 16B. This observation stems from the fact that as the air pressure within the air cannon increases, the airflow velocity augments, intensifying its impact on the soil. Concurrently, the airflow’s diffusion range expands, leading to a pronounced soil cracking effect that facilitates easier soil loosening.

According to the standards for evaluating subsoiling operations, the soil looseness after subsoiling should fall within a range of 10% to 40%, while the soil disturbance coefficient must not drop below 50% [51]. The experimental values for soil looseness and soil disturbance coefficient were calculated using equations (8) and(9) to evaluate the subsoiling effect. From Table 5, it can be observed that the working air pressure has a significant impact on the soil disturbance area during the air blast subsoiling process. However, no clear linear correlation emerges between the working air pressure and either soil looseness or the soil disturbance coefficient. Specifically, at an air pressure of 0.6 MPa, both soil looseness and the soil disturbance coefficient surpass their respective values at 0.4 MPa and 0.8 MPa. The field experimental conditions introduce additional complexity, given the unevenness of the soil surface and the presence of crop residue, which may influence data accuracy. Overall, the supersonic gas jet subsoiler demonstrates significant subsoiling effects and meets the relevant evaluation standards.

**Table 5:** Experimental results of soil loosening degree and soil disturbance coefficient.

Pressure (MPa)	A_h_ (cm^2^)	A_q_ (cm^2^)	A_s_ (cm^2^)	soil looseness (%)	Soil disturbance coefficient (%)
0	1303.4	1140	590.2	14.33	51.77
0.4	1299.1	1140	612	13.96	53.68
0.6	1352.3	1178	664.3	14.8	56.39
0.8	1481.1	1292	722.9	14.64	55.95
Average	1358.98	1187.5	647.35	14.44	54.51

## Conclusion

This study introduces an innovative method for gas explosion subsoiling reduction draft force by integrating an air cannon with the standard wing-shaped subsoiler structure, thereby leading to the design of the supersonic gas jet subsoiler. Through theoretical analysis, mathematical equations were derived to delineate the gas explosion parameters of the subsoiler. Furthermore, soil bin experiments were conducted to verify the feasibility of utilizing the air cannon for soil pressure splitting. Leveraging the EDEM software, a soil model was developed that mirrored actual field soil conditions, enabling a comprehensive analysis of the subsoiling process and the stress condition acting on the subsoiler. These findings were subsequently corroborated through field experiments. The research culminated in three key conclusions.

(1) The soil bin experimental results indicate that the high-velocity airflow generated by the air cannon effectively fractures the soil, leading to surface uplift and the formation of numerous cracks. The amount of soil uplift and the extent of crack propagation caused by air pressure are linearly correlated with the air pressure, and both decrease with increasing horizontal distance and decreasing air pressure. Therefore, the application of air cannon technology in subsoiling operations is feasible.
(2) The analysis of the tillage process of the subsoiler under nine diverse operating conditions, conducted using EDEM software, has generated insightful results. The forces acting on the supersonic gas jet subsoiler are predominantly concentrated on the tip, wing, and shank of the blade, with the highest force intensity recorded at the tip. Notably, minimal force was detected on the spray nozzle, and there was an absence of any significant particle accumulation between the nozzle and the blade shank, which reduced the contact area and adhesion between the soil particles and the nozzle. A comparative assessment of the draft resistance between two types of subsoilers, under identical working conditions, revealed that the draft force of the supersonic gas jet subsoiler did not undergo a substantial increase. This finding suggests that the installation of the nozzle on the winged subsoiler did not compromise its operational performance.
(3) The field experimental results show that under different working conditions, the designed supersonic gas jet subsoiler demonstrated a remarkable reduction in draft resistance, ranging from 2.21% to 16.66%. Simultaneously, it enhanced the soil disturbance area by 3.69%−22.48%. Additionally, as the working speed decreased, the frequency of gas explosions per unit working distance increased, leading to a notable enhancement in the subsoiling efficacy. The soil looseness achieved was within the range of 13.96% to 14.8%, while the soil disturbance coefficient ranged from 53.68% to 56.39%, aligning with the established evaluation standard for subsoiling operations. Furthermore, no discernible linear correlation was observed between the working air pressure and either the soil looseness or the soil disturbance coefficient.

The results of this study provide a novel approach for achieving subsoiling through the application of high-pressure gas. Experimental findings show that the supersonic gas jet subsoiler effectively fulfills the criteria for subsoiling, with the optimal subsoiling performance achieved at a working speed of 0.5 m/s, a working depth of 380 mm, and a pressure setting of 0.8 MPa.

## Supporting information

S1 File Distance Calculation. (DOCX)

S2 File Take pictures. (DOCX)
