# Supplementary material for: Numerical simulation and field experiment study of the supersonic gas jet subsoiler based on DEM
Source: PLoS One. 2025 Aug 14;20(8):e0328565. doi: 10.1371/journal.pone.0328565 (PMC12352846; doi:10.1371/journal.pone.0328565)
Supplement: S1 File — (DOCX) [file pone.0328565.s001.docx]

**Distance Calculation**

# -*- coding: utf-8 -*-
import pyrealsense2 as rs
import numpy as np
import cv2
import math
import matplotlib.pyplot as plt

'''
设置
'''
pipeline = rs.pipeline() # 定义流程pipeline，创建一个管道
config = rs.config() # 定义配置config
config.enable_stream(rs.stream.depth, 1280, 720, rs.format.z16, 30) # 配置depth流
config.enable_stream(rs.stream.color, 1280, 720, rs.format.bgr8, 30) # 配置color流

pipe_profile = pipeline.start(config) # streaming流开始

# 创建对齐对象与color流对齐
# align_to = rs.stream.color # align_to 是计划对齐深度帧的流类型
# align = rs.align(align_to) # rs.align 执行深度帧与其他帧的对齐
align = rs.align(rs.stream.color) #将上两句合成一句，将深度与color对齐

'''
获取对齐图像帧与相机参数
'''


def get_aligned_images():
 frames = pipeline.wait_for_frames() # 等待获取图像帧，获取颜色和深度的框架集
 aligned_frames = align.process(frames) # 获取对齐帧，将深度框与颜色框对齐

 aligned_depth_frame = aligned_frames.get_depth_frame() # 获取对齐帧中的的depth帧
 aligned_color_frame = aligned_frames.get_color_frame() # 获取对齐帧中的的color帧

 #### 获取相机参数 ####
 depth_intrin = aligned_depth_frame.profile.as_video_stream_profile().intrinsics # 获取深度参数（像素坐标系转相机坐标系会用到）
 color_intrin = aligned_color_frame.profile.as_video_stream_profile().intrinsics # 获取相机内参

 #### 将images转为numpy arrays ####
 img_color = np.asanyarray(aligned_color_frame.get_data()) # RGB图
 img_depth = np.asanyarray(aligned_depth_frame.get_data()) # 深度图（默认16位）

 return color_intrin, depth_intrin, img_color, img_depth, aligned_depth_frame


'''
获取随机点三维坐标
'''


def get_3d_camera_coordinate(depth_pixel, aligned_depth_frame, depth_intrin):
 x = depth_pixel[0]
 y = depth_pixel[1]
 dis = aligned_depth_frame.get_distance(x, y) # 获取该像素点对应的深度
 # print ('depth: ',dis) # 深度单位是m
 camera_coordinate = rs.rs2_deproject_pixel_to_point(depth_intrin, depth_pixel, dis)
 # print ('camera_coordinate: ',camera_coordinate)
 return dis, camera_coordinate


if __name__ == "__main__":
 while True:
 '''
 获取对齐图像帧与相机参数
 '''
 color_intrin, depth_intrin, img_color, img_depth, aligned_depth_frame = get_aligned_images() # 获取对齐图像与相机参数

 '''
 获取随机点三维坐标
 '''
 xx1=346
 yy1=634
 depth_pixel1 = [xx1, yy1] # 设置随机点，以相机中心点为例320、240
 dis1, camera_coordinate1 = get_3d_camera_coordinate(depth_pixel1, aligned_depth_frame, depth_intrin)


 xx2=384
 yy2=393
 depth_pixel2 = [xx2, yy2] # 设置随机点，以相机中心点为例320、240
 dis2, camera_coordinate2 = get_3d_camera_coordinate(depth_pixel2, aligned_depth_frame, depth_intrin)


 '''
 显示图像与标注
 '''
 #### 在图中标记随机点及其坐标 ####
 cv2.circle(img_color, (xx1, yy1), 3, [0, 255, 0], thickness=1)
 cv2.circle(img_color, (xx1, yy1), 6, [0, 255, 0], thickness=1)
 cv2.putText(img_color, "Dis1:" + str(dis1) + " m", (40, 40), cv2.FONT_HERSHEY_SIMPLEX,0.5, [0, 0, 255])
 cv2.putText(img_color, "X1:" + str(camera_coordinate1[0]) + " m", (40, 60), cv2.FONT_HERSHEY_SIMPLEX, 0.5,
 [255, 0, 0])
 cv2.putText(img_color, "Y1:" + str(camera_coordinate1[1]) + " m", (40, 80), cv2.FONT_HERSHEY_SIMPLEX, 0.5,
 [255, 0, 0])
 cv2.putText(img_color, "Z1:" + str(camera_coordinate1[2]) + " m", (40, 100), cv2.FONT_HERSHEY_SIMPLEX, 0.5,
 [255, 0, 0])
 cv2.putText(img_color, "1", (xx1-5, yy1-9), cv2.FONT_HERSHEY_SIMPLEX, 0.5,[0, 255, 0])

 cv2.circle(img_color, (xx2, yy2), 3, [255, 0, 255], thickness=1)
 cv2.circle(img_color, (xx2, yy2), 6, [255, 0, 255], thickness=1)
 cv2.putText(img_color, "Dis2:" + str(dis2) + " m", (350, 40), cv2.FONT_HERSHEY_SIMPLEX,0.5, [0, 0, 255])
 cv2.putText(img_color, "X2:" + str(camera_coordinate2[0]) + " m", (350, 60), cv2.FONT_HERSHEY_SIMPLEX, 0.5,
 [255, 0, 0])
 cv2.putText(img_color, "Y2:" + str(camera_coordinate2[1]) + " m", (350, 80), cv2.FONT_HERSHEY_SIMPLEX, 0.5,
 [255, 0, 0])
 cv2.putText(img_color, "Z2:" + str(camera_coordinate2[2]) + " m", (350, 100), cv2.FONT_HERSHEY_SIMPLEX, 0.5,
 [255, 0, 0])
 cv2.putText(img_color, "2", (xx2 - 5, yy2 - 9), cv2.FONT_HERSHEY_SIMPLEX, 0.5, [255, 0, 255])

 cv2.line(img_color, (xx1,yy1), (xx2,yy2), [0, 255, 255], 1)
 if camera_coordinate1[0]*camera_coordinate1[1]*camera_coordinate1[2]*camera_coordinate2[0]*camera_coordinate2[1]*camera_coordinate2[2]==0:
 cv2.putText(img_color, "Dis1to2:" + "Please select points with depth", (40, 150), cv2.FONT_HERSHEY_SIMPLEX, 1, [0, 255, 255])
 else:
 juli=math.sqrt((camera_coordinate2[0]-camera_coordinate1[0])**2+(camera_coordinate2[1]-camera_coordinate1[1])**2+(camera_coordinate2[2]-camera_coordinate1[2])**2)
 cv2.putText(img_color, "Dis1to2:" + str(juli) + " m", (40, 150), cv2.FONT_HERSHEY_SIMPLEX, 1, [0, 255, 255])

 ### 显示画面 ####
 cv2.imshow('RealSence', img_color)
 key = cv2.waitKey(1)
 if key & 0xFF == ord('q') or key == 27:
 cv2.destroyAllWindows()
 break
