# Supplementary material for: Numerical simulation and field experiment study of the supersonic gas jet subsoiler based on DEM
Source: PLoS One. 2025 Aug 14;20(8):e0328565. doi: 10.1371/journal.pone.0328565 (PMC12352846; doi:10.1371/journal.pone.0328565)
Supplement: S2 File — (DOCX) [file pone.0328565.s002.docx]

**Take pictures**

import pyrealsense2 as rs
import numpy as np
import cv2

counter = 0
# 若要保存到其他文件夹，修改此路径
folder = 'C:/ZZP XZ/Engineering software/study/1/'


def shot(pos, frame):
 global counter
 path = folder + pos + "_" + str(counter) + ".png"
 cv2.imwrite(path, frame)
 print("snapshot saved into: " + path)


pipeline = rs.pipeline()
config = rs.config()
# 配置深度和颜色流
# 10、15或者30可选,20或者25会报错，其他帧率未尝试
# 配置颜色相机
config.enable_stream(rs.stream.color, 1280, 720, rs.format.bgr8, 30)
# 配置红外相机
config.enable_stream(rs.stream.infrared, 1, 1280, 720, rs.format.y8, 30)
config.enable_stream(rs.stream.infrared, 2, 1280, 720, rs.format.y8, 30)
# 配置深度图像
config.enable_stream(rs.stream.depth, 1280, 720, rs.format.z16, 30)
# Start streaming
profile = pipeline.start(config)

# 创建对齐对象, rs.align 允许我们将深度帧与其他帧对齐, "align_to" 是计划对其深度帧的流类型
align_to = rs.stream.color
align = rs.align(align_to)

try:
 while True:
 frames = pipeline.wait_for_frames()
 # 将深度框与颜色框对齐
 aligned_frames = align.process(frames)
 # 获取对齐帧
 aligned_depth_frame = aligned_frames.get_depth_frame()
 if not aligned_depth_frame:
 continue
 depth_frame = 50 * np.asanyarray(aligned_depth_frame.get_data())
 # 将深度图转化为伪彩色图方便观看
 depth_colormap = cv2.applyColorMap \
 (cv2.convertScaleAbs(depth_frame, alpha=0.008)
 , cv2.COLORMAP_JET)
 cv2.imshow('1 depth', depth_colormap)

 # color frames
 color_frame = aligned_frames.get_color_frame()
 if not color_frame:
 continue
 color_frame = np.asanyarray(color_frame.get_data())
 cv2.imshow('2 color', color_frame)

 # left　frames
 left_frame = frames.get_infrared_frame(1)
 if not left_frame:
 continue
 left_frame = np.asanyarray(left_frame.get_data())
 cv2.imshow('3 left_frame', left_frame)

 # right framesq
 right_frame = frames.get_infrared_frame(2)
 if not right_frame:
 continue
 right_frame = np.asanyarray(right_frame.get_data())
 cv2.imshow('4 right_frame', right_frame)

 c = cv2.waitKey(1)

 # 如果按下ESC则关闭窗口（ESC的ascii码为27），同时跳出循环
 if c == 27:
 cv2.destroyAllWindows()
 break

 if c == ord('t'):
 # 默认保存五张图片，如果不需要保存某图像把对应那行代码注释掉即可
 shot('left_shot', left_frame)
 shot('right_shot', right_frame)
 shot('color_shot', color_frame)
 shot('depth_colormap', depth_colormap)
 shot('depth_frame', depth_frame)
 counter += 1

finally:
 # Stop streaming
 pipeline.stop()
